# Brain Drain in Cancer Care: The Shrinking Clinical Oncology Workforce in Nigeria

**DOI:** 10.1200/GO.23.00257

**Published:** 2023-12-21

**Authors:** Runcie C.W. Chidebe, Tochukwu C. Orjiakor, Nwamaka Lasebikan, Adedayo Joseph, Samantha Toland, Alison Simons

**Affiliations:** ^1^ Project PINK BLUE- Health & Psychological Trust Centre, Abuja, Nigeria; ^2^ Faculty of Health, Education & Life Sciences, Birmingham City University, Birmingham, United Kingdom; ^3^ Department of Sociology & Gerontology, Miami University, Miami, OH; ^4^ Scripps Gerontology Center, Miami University, Miami, OH; ^5^ Department of Psychology, University of Nigeria, Enugu, Nigeria; ^6^ Department of Psychology, University of Toronto, Scarborough, Canada; ^7^ Health Policy Research Group, Department of Pharmacology and Therapeutics, College of Medicine, University of Nigeria, Enugu, Nigeria; ^8^ Oncology Center, University of Nigeria Teaching Hospital, Enugu, Nigeria; ^9^ Association of Radiation & Clinical Oncologists in Nigeria (ARCON), Abuja, Nigeria; ^10^ NSIA—LUTH Cancer Center, Lagos University Teaching Hospital, Lagos, Nigeria; ^11^ The West Midlands Cancer Alliance SACT (Systemic Anti-Cancer Treatment) Expert Advisory Group, Worcester, United Kingdom; ^12^ Worcestershire Acute Hospital NHS Trust, Worcester, United Kingdom

## Abstract

**PURPOSE:**

A recent estimate indicates that Nigeria has about 70 clinical oncologists (COs) providing care for 124,815 patients with cancer and its 213 million total population. This staggering deficit is likely to worsen as about 90% of Nigerian physicians are eager to leave the country for perceived greener pastures in the United States, the United Kingdom, Canada, etc. Previous studies have examined general physician migration abroad; however, the CO workforce in Nigeria has been barely considered in the workforce literature. This study examined the push and pull factors to stay or leave the CO workforce and Nigeria.

**METHODS:**

Using a correlational design, 64 COs completed turnover intention (TI), workload, and satisfaction measures. Multiple linear regression was used for the data analysis.

**RESULTS:**

The results show that CO workload (number of outpatients attended to; *r* = 0.30, *P* < .01) and satisfaction with the delivery of CO care (*r* = 0.23, *P* < .05) were significantly related to TI. The number of outpatients seen was also positively linked to TI. Hence, the more outpatients a CO sees, the higher the intention to leave. The United States (31%), the United Kingdom (30%), and Canada (10%) were the top countries of destinations for Nigerian COs.

**CONCLUSION:**

Higher CO workload is a push factor propelling the intention to leave CO practice and relocate to other countries. Nigeria's new National Cancer Control Plan and the Federal Ministry of Health need to explore innovative approaches to attract and retain the CO workforce, which would lead to improvement in cancer survival and outcomes. Increasing the number of CO programs and positions available, improving work conditions, and introducing work benefits may mitigate the shrinking CO workforce in Nigeria.

## Teaser

Only 80 oncologists care for over 120,000 patients in Nigeria, yet the country is losing its oncologists to high-income countries. The United States, the United Kingdom, and Canada are top destinations. What factors are responsible for this brain drain?

## INTRODUCTION

The shortage of the oncology workforce in sub-Saharan Africa (SSA) is likely to determine the pace of scaling up cancer care in the region because any investment made in cancer without comprehensively addressing shortfalls in the oncology workforce will fail.^1^ Of the eight countries that had no clinical oncologists (COs) to provide care for patients with cancer, seven are in Africa.^2^ The WHO posited that there is no health without a workforce.^3^ The health care workforce is so important that the best and most expensive equipment is not sufficient to make up for gaps in the workforce needed to operate them. If any country has all the best medical machines without the workforce to operate those medical machines, the health system will be largely lacking and may not achieve its potential.^4^

**CONTEXT**

**Key Objective**
There are only 80 clinical oncologists (COs) providing care for more than 120,000 patients in Nigeria, yet the country is losing its oncologists to high-income countries. What factors are responsible for this brain drain in cancer care?
**Knowledge Generated**
The higher the clinical workload, the higher the COs' intention to leave the workforce. The more time spent attending to outpatients, the higher their intention to stay. Despite workforce shortage, most COs intend to leave Nigeria; the United States, the United Kingdom, and Canada are top destinations. Empathy for patients with cancer was a key attraction to oncology for COs.
**Relevance**
Higher clinical workload impacts patients' treatment outcomes; hence, the country may not improve cancer survival rates without improving the workforce. Nigerian government should improve working conditions, invest in postgraduate colleges, and increase remuneration. A government-to-government agreement needs to be explored to get destination countries to invest in medical school training in Nigeria.

COs are physicians who use radiotherapy and chemotherapy/systemic therapies to manage patients with cancer,^5^ whereas medical oncologists are physicians who use chemotherapy/systemic therapy to manage patients with cancer.^6^ The ratio of CO per new patients with cancer was 1 to 1,000 patients in 25 African countries, and the mortality-to-incidence ratio was >70% in 21 African countries.^2^ Although the ratio of CO per new cancer case is 476 per 689 new patients with cancer in the United Kingdom; 11,700:137 in the United States; 517:352 in Canada; and 448:272 in Australia, in Nigeria, it is 26 oncologists per 3,923.^2^ For a population of more than 213 million,^7^ Nigeria has only four medical doctors per 10,000 patients in 2021^8^ and 15 nurses and midwives per 10,000 patients in 2019.^9^ Yet, 9 in 10 Nigerian physicians are seeking opportunities to leave for the United States, the United Kingdom, and Canada.^10^

With the increasing cancer incidence and mortality, Nigeria is facing shortages of the oncology workforce to care for the population diagnosed with cancer in different parts of the country fueled by brain drain.^11,12^ Brain drain is the migration of skilled and educated workforce from less developed regions or countries to more economically established ones.^13^ The effect of brain drain and the resultant shortage is stark in different subspecialties of oncology in SSA. Although many studies on workforce migration have been conducted among general physicians, the push and pull factor for brain drain in cancer care has remained unclear and understudied. The available data investigating CO workforce migration has been mostly broad-based, covering the entire African continent and not specific to Nigeria.^2,14,15^ Understanding the in-country factors and dynamics that contribute to specific workforce-type brain drain can inform policymakers, Nigeria's Federal Ministry of Health and Labor, the National Institute for Cancer Research and Treatment (NICRAT), and other global actors to respond better to the impending clinical oncology workforce crisis. The aim of this study was to investigate the push and pull factors (turnover intention [TI], ie, intention to stay or leave a workplace) in the CO workforce in Nigeria. It was hypothesized that there would be a significant association between CO workload, satisfaction in the delivery of CO care (SDCOC), and TI among the CO workforce in Nigeria.

## METHODS

### Participants

All COs registered with the national professional society, known as the Association of Radiation and Clinical Oncologists of Nigeria (ARCON), were targeted for this study. The COs were invited to participate in the study through the ARCON secretariat. The target was to capture all the COs in Nigeria; hence, 80 COs participated, where 64 were included in the main study and 16 in the pilot study. Thus, 64 COs working in oncology facilities located across 17 states in all six geopolitical regions of Nigeria including the Abuja Federal Capital Territory (FCT) were part of this study. The exact number of COs in Nigeria is not officially documented by any agency; however, a recent study reported that Nigeria has <70 COs.^16^ Hence, this study's sample size of 64 is appropriate. Only COs, that is physicians who use radiotherapy and systemic therapy to treat patients with cancer in Nigeria, were included in the study.^5^ Other oncology professionals such as general physicians, pathologists, nurses, radiographers, and medical physicists who provide oncology care services in Nigeria were excluded. Nigerian COs working abroad were also excluded from this study.

### Measures

#### Instruments

Three instruments were used in this study to measure TI, CO workload, and satisfaction in the delivery of oncology care. A section of the survey form was used to obtain the demographic characteristics of participants. The turnover intention scale (TIS) used in this study was a 17-item scale adapted from Roodt's (2004) 15-item TIS^17^ and TIS-12.^18^ TIS has been found to have an acceptable level of internal consistency reliability with a Cronbach alpha of .70^18^ and .80.^19^ For TIS to have response reliability, researchers should have respondents indicate their responses to TIS within a reasonable timeframe.^20^ Respondents are thus required to indicate their intention to leave or stay at their workplace in the past 9 months. The global oncology workload survey (GOWS) developed by Fundytus et al^21^ was adapted and used to measure CO workload. GOWS is 50-item survey that has been found to be reliable and used in several studies on CO workload in many countries.^15,22^ The researchers developed SDCOC.

#### Reliability and Validity

In this study, a pilot study (see Appendix) was conducted on the instruments, and reliability and validity were as follows: (1) The item analysis on the TIS showed a Cronbach alpha of .71 and (2) the SDCOC showed α = .76. The Cronbach alpha had good reliability indices and the test for normal distribution on the core variables of concern, and scores were normally distributed, hence permitting the use of probability statistics.^23^ Items 1-3 mandated participants to give their consent before they could proceed to the actual questionnaire. Items 4-7 asked participants to provide demographics such as age, sex, marital status, and location of practice. Items 8 to 16 measured CO workload. Items 17 (17a, b, c, d, and e) and 18 measured satisfaction with delivering CO care. Items 19 to 40 were TIS. Items 34, 35, and 38 were reversed-scored since they were negatively worded. Items 29, 30, 34, 35, and 37 were adjusted to suit the CO setting. Items 19, 22, 23, 31, 32, 33, 39, and 40 were new items. Items 19 and 40 were open-ended.

#### Data Collection

The data collection was through the Birmingham City University–approved survey platform known as Online Survey. The researchers shared the questionnaire link with the ARCON secretariat, and it was sent to all ARCON members through emails, short message service, and WhatsApp messages across the country to ensure diversity, equity, and inclusion. All participants who volunteered to participate in this study were given the participants' information sheet and accepted to participate voluntarily by clicking on the “I accept” on the consent page for the questionnaire. No participant was allowed to proceed to the questionnaire section without giving consent.

### Data Analysis

The responses were coded into IBM Statistical Package for the Social Sciences (SPSS) Statistics version 24.0 (IBM Corp, Armonk, NY) for data analysis.^24^ Considering that this study wanted to estimate the relationship between two independent variables (CO workload and satisfaction in the delivery of CO care) on a dependent variable (TI). A multiple regression model (stepwise) was used to build a model for predicting TI. The control variables, sex, age, and marital status were put in the first step. The various indicators of CO workload were added in the second step, and satisfaction with delivering CO care was used in the third step. Content analysis was used to analyze the open-ended item responses and grouped them into categories.

### Ethics

This study was approved by the Birmingham City University Health, Education, and Life Sciences Faculty Academic Ethics Committee (Chidebe/7454/R(C)/2020/Jul/HELSFAEC); University of Nigeria Teaching Hospital Health Research Ethics Committee (NHREC/05/01/2008B-FWA0002458-1RB00002323); and Lagos University Teaching Hospital Health Research Ethics Committee (ADM/DCST/HREC/APP/3719).

## RESULTS

### Participant Demographic Characteristics

Table 1 shows the demographic characteristics of the participants. Of the 64 participants who completed the questionnaire, 48 (75%) were male and 16 (25%) were female; 54 (84.4%) were married, nine (14.1%) were single, and one (1.6%) was widowed.

**TABLE 1:**
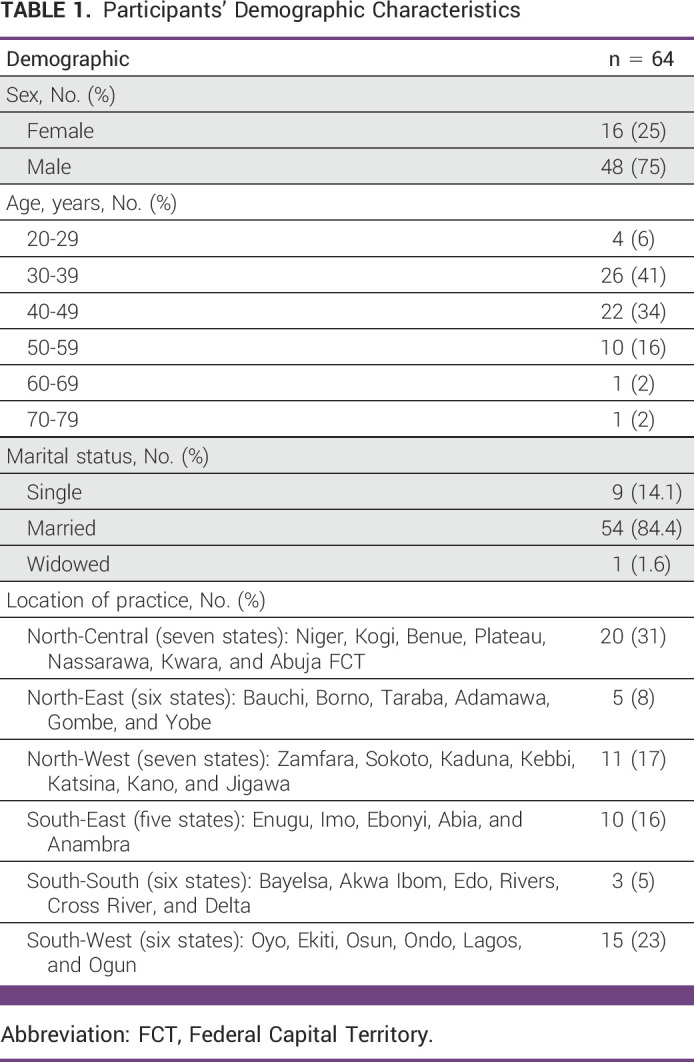
Participants' Demographic Characteristics

### Turnover Intention in Clinical Oncologists Across the Geopolitical Zones and Participants' Distribution by Sex

**FIG 1:**
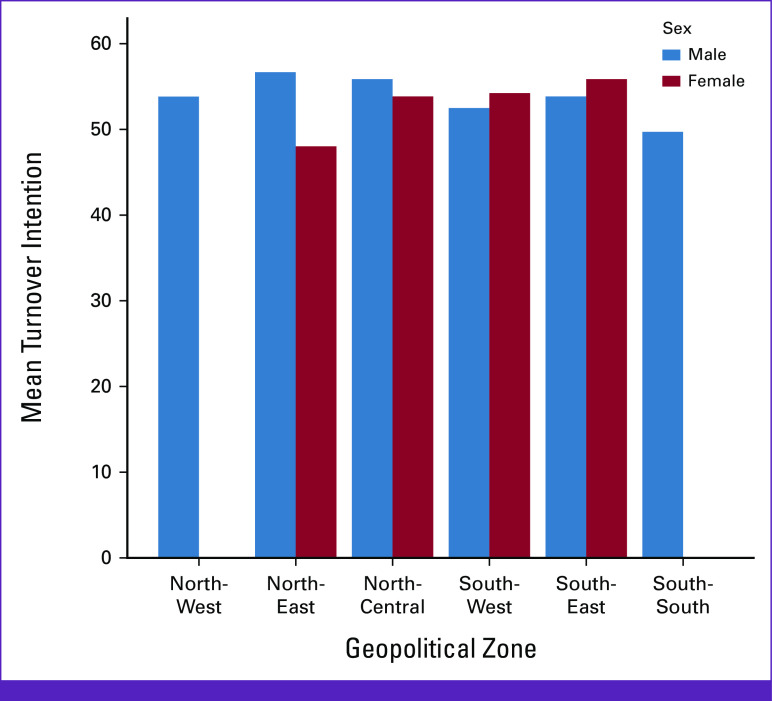
Showing the mean turnover intention of participants by geopolitical zones and sex.

Figure 1 shows the mean TI of participants by geopolitical zones and sex. TI was also calculated across geopolitical zones in Nigeria, and most of the participants' scores were above 50, whereas the mean score was 47.5 (the mean score was calculated as follows: 19 questions with a possible total mark of 95, hence 95 divided by 2 equals the mean of 47.5). Therefore, there is an inclination to TI among all the participants. Hence, the participants had the inclination to leave the CO workforce and Nigeria. Participants in the North-East and South-East had the highest inclination to leave the CO workforce or leave Nigeria.

**FIG 2:**
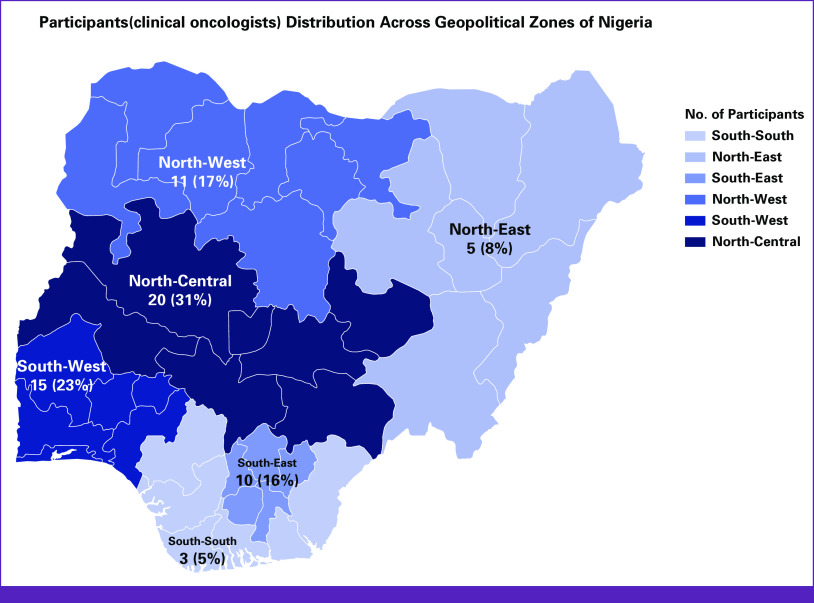
Participants distribution across the country.

The findings suggest that it is most likely that Nigeria does not have more than 80 practicing COs. Furthermore, the results indicate that Abuja FCT has the highest number of COs, followed by Lagos and Kaduna states. For geopolitical regions, the North-Central is most likely to have the highest number of COs, followed by the South-West, North-West, and South-East. The South-South region is most likely to have the lowest number of COs in Nigeria (Table 1 and Fig 2). On the basis of participants' sex, the North-Central and South-East of Nigeria are most likely to have the highest number of female COs, followed by the South-West. North-East, North-West, and South-South have the likelihood of not having or having the lowest number of female COs.

### Preferred Destination for TI in CO Workforce

**FIG 3:**
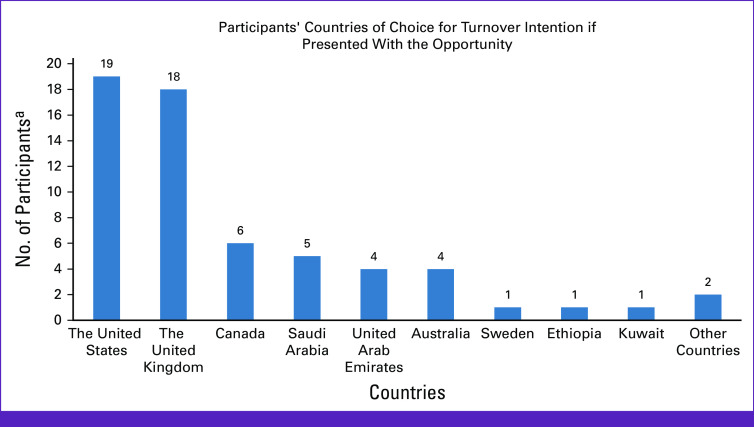
Participants' countries of choice for turnover intention if presented with the opportunity. ^a^Only 61 responses were recorded from the 64 participants.

Figure 3 shows participants' preferred destination/country of choice when presented with the opportunity to leave Nigeria. The United States (31%)^19^ was the most preferred country of destination for TI among the participants while the United Kingdom (30%)^18^ was second most preferred, followed by Canada (10%),^6^ Saudi Arabia (8%),^5^ United Arab Emirates (7%),^4^ and Australia (7%)^4^. Kuwait, Sweden, Ethiopia, and other countries had the lowest scores (8%)^5^ as destinations of choice if participants were presented with the opportunity to leave Nigeria.

### Choice of Clinical Oncology Over Other Areas of Medicine

**FIG 4:**
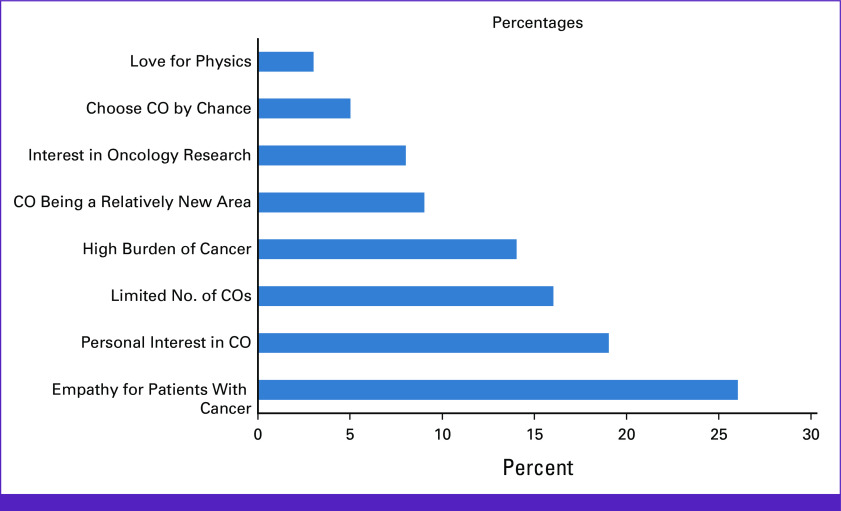
Shows the reasons for choosing clinical oncology over other areas of medicine. CO, clinical oncologist.

The finding shows that Nigeria COs were pulled into the CO workforce for different reasons. Participants reported having chosen CO over other areas of medicine because of empathy for patients with cancer (26%),^17^ personal interest in CO (19%),^12^ the limited number of COs (16%),^10^ the high burden of cancer (14%),^9^ CO being a relatively new area of medicine (9%),^6^ interest in oncology research (8%),^5^ chose CO by chance (5%),^3^ and love for physics (3%; Fig 4).^2^

### Correlation of Sex, Age, Marital Status, CO Workload, and SDCOC with TI

Table 2 shows some interesting significant relationships. CO workload (number of outpatients attended to; *r* = 0.30, *P* < .01) and satisfaction with the delivery of clinical oncology care (*r* = 0.23, *P* < .05) were significantly related to TI. Satisfaction with the delivery of clinical oncology care was also significantly related to age (*r* = −0.36, *P* < .01) and clinical oncology workload (number of outpatients attended to; *r* = 0.32, *P* < .01).

**TABLE 2:**
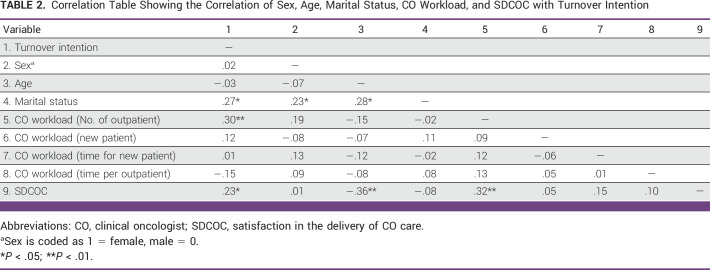
Correlation Table Showing the Correlation of Sex, Age, Marital Status, CO Workload, and SDCOC with Turnover Intention

### Regression on Sex, Age, Marital Status, CO Workload, SDCOC, and TI

Table 3 shows that CO workload (number of outpatients seen; β = .29) and time spent on outpatients (β = −.23) significantly predicted TI. The number of outpatients seen was also positively linked to TI. Hence, the more outpatients a CO sees, the higher the TI. An unexpected finding was the inverse relationship between time spent attending to oncology outpatients (CO consultations) and TI. Interestingly, the more time spent attending to oncology outpatients, the lower the TI.

**TABLE 3:**
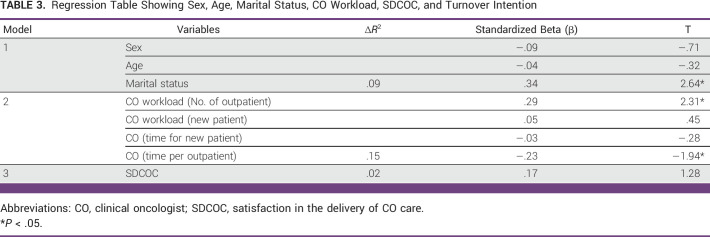
Regression Table Showing Sex, Age, Marital Status, CO Workload, SDCOC, and Turnover Intention

## DISCUSSION

Although there are studies that have explored brain drain among medical practitioners in Nigeria, studies focusing on COs are scarce. At the time of writing, we did not find any literature describing the factors pulling and pushing COs in and out of the CO workforce in Nigeria. Yet in Africa, the burden of cancer is the highest in Nigeria, and the proportion of COs serving the most populous country in Africa is poor and further threatened by numerous forces. Understanding the dynamics of the available CO workforce is key to improving service delivery and health outcomes in the country.^1,25,26^

To our knowledge, this is the first study investigating the push and pull factors in the CO workforce in Nigeria and possibly the first study using a correlative design and statistics to explain CO brain drain in SSA. Some significant findings emerged from this study. We found that CO workload and time spent on outpatients significantly predicted TI. The number of outpatients seen was also positively linked to TI. Hence, the more outpatients a CO sees, the higher the TI. This finding is consistent with those of previous studies whose results showed that high CO workload is a push factor increasing the number of African oncologists willing to leave their local CO workplaces to other regions and a reason for fewer physicians willing to specialize in CO.^15^ This study's central finding appears to show that high CO workload could affect patients' treatment outcome. The finding is interesting in showing the association between CO workload and TI. Our findings may imply that Nigerian COs have a high inclination to leave their CO practice in Nigeria.

An unexpected finding was that the amount of time spent attending to oncology outpatients was inversely related to TI. Thus, the more time spent attending to oncology outpatients, the lower the intention to leave. It means that more time spent in care means that the COs value the time spent with patients and thus are more satisfied with their work. The participants seem to be happier spending time with patients. The time they have with patients contributes to them not intending to leave the CO workforce and Nigeria. This finding seems to be an indication of the passion that COs put into their work. The more time and commitment they give to their patients, the more they are attracted to staying in their local setting to keep delivering CO care.

We also found that SDCOC was significantly related to TI. Hence, the lower the level of SDCOC, the higher the TI. This study is also consistent with the studies that reported satisfaction as being significantly associated with TI.^27,28^ In our study, satisfaction is the level of contentedness and affective reaction in giving cancer care in comparison with the real, desired, and deserved results; hence, it appears that many push factors increasingly decrease satisfaction. Therefore, our study suggests that regular evaluation should be carried out among COs in Nigeria to learn and understand how to continue to improve their SDCOC to patients while reducing TI with an understanding that satisfaction is subjective.

This study subtly corroborates the notion that COs are very willing to leave their current working positions as COs and the country to seek a better living elsewhere. Developed economies that are able and willing to offer higher wages, better working conditions, and better health care systems seem to be more attractive to Nigerian COs. The United States, the United Kingdom, and Canada are top destinations of interest. Our finding is in congruity with a survey that reported that 9 in 10 Nigerian physicians are seeking opportunities abroad,^10^ and that the United States, the United Kingdom, and Canada are top destinations.^29-33^ This study's results appear to explain that physicians' migration from low- and middle-income countries (LMICs) such as Nigeria to high-income countries (HICs) such as the United States, the United Kingdom, and Canada affects Nigeria in diverse ways. For instance, the mortality cost of Nigerian physician migration to HICs totals $3.1 billion US dollars (USD) annually.^29^ The Nigerian government loses at least N3.8 million ($4,798 USD) for subsidizing the training of its physicians who eventually leave the country to HICs.^34^ Since the United Kingdom, the United States, Canada, and other HICs did not provide medical school training to those physicians who emigrated from Nigeria, Africa, and other LMICs, the United Kingdom saves over £1.9 billion ($2.7 billion USD), the United States over $4.5 billion USD and Canada over $384 million USD for not training physicians that they pulled to their countries.^34^ The Nigeria Medical Association (NMA) reports that Nigeria has 74,543 registered physicians; however, only an estimated 40,000 are practicing in the country for a population of 213 million.^35,36^ Although 24% of the world disease burden is in Africa coupled with physician migration and the recent COVID-19 pandemic,^37^ the UK Home Office is relaxing its immigration policies to attract more non-British doctors to the United Kingdom.^38^

The implication of this study's finding is that if brain drain in cancer care remains unchecked, access to timely treatment would be worse, and patient outcomes would significantly become poorer. We propose the following policy recommendations. First, Nigeria's Ministries of Health and Labor should improve working conditions, invest in postgraduate colleges, upgrade treatment centers, increase remuneration and payment of arrears, reduce CO workload by recruiting COs, and introduce workplace benefits like low-interest loans (ie, car loans), housing support, and other personal benefits. Second, there is an urgent need for multi-government engagement. The Nigerian government needs to engage with HIC governments to develop a mutually beneficial physician migration policy that would get the destination countries—the United Kingdom, the United States, Canada, and others—to invest in medical school training in Nigeria. Third, the WHO needs to engage with HICs to honor their commitment to the WHO Health Workforce Support and Safeguards List 2023—a list of 55 LMICs (including Nigeria) facing the most pressing health workforce challenges. This WHO policy discourages HICs from active recruitment of health workforce from those 55 LMICs unless there are government-to-government (G2G) agreements.^39^ For instance, as of 2023, there is no known G2G agreement between the United Kingdom and Nigeria; however, the United Kingdom has continued to actively recruit from Nigeria. Fourth, HICs should fund global oncology hands-on training, skills, and knowledge exchange programs such as the Upgrade Oncology: US–Nigeria science and technology exchange program using the Fulbright Specialists model and implemented by Project PINK BLUE.^25^ Finally, the Tertiary Education Trust Fund, NICRAT, and NMA should collaboratively raise funds to offer generous research funding to COs. This strategy helped Thailand and Ireland in reversing brain drain.^40^

The use of a correlational design was appropriate for this study; however, causality cannot be established. On the other hand, one of the most important limitations of this study is the choice of study participants, who are practicing COs in Nigeria. It is important to note that a deeper understanding of pull factors in the CO workforce may be better explored with participants such as medical students, fresh graduates of MBBS, or residents who are yet to choose a specialty. Future studies on push and pull factors in clinical oncology should also include medical students, fresh graduates of MBBS, residents who are yet to choose a specialty, or recent residents in the West African College of Surgeons' faculty of radiology. COs who have already left Nigeria would also provide interesting insights on brain drain. It may also be interesting for future studies to use qualitative research methods and conduct comparative analyses of push and pull factors. The researchers are currently looking for grants to study Nigerian COs who already moved abroad and to expand the study participants to SSA.

In conclusion, brain drain in cancer care is possibly one of the most pressing challenges of cancer control in Nigeria. Higher CO workload is one of the leading factors propelling the intention to leave CO practice and relocate to other countries. More COs can be available for employment when they are attracted, and their training is optimized. As a country with the highest cancer burden in Africa, the country needs urgent intervention by the government, international/local nongovernmental organizations, private sectors, and other stakeholders. Nigeria's Federal Ministry of Health and Labour and NICRAT should consider applying our policy recommendations in attracting, retaining, and mitigating brain drain, which would lead to improvement in cancer survival and outcomes.

## APPENDIX. PILOT STUDY

A pilot study was conducted using the questionnaire to check feasibility, validity, reliability, removal of flaws, and verify useable data through analysis.^37^ The questionnaire was sent to Association of Radiation and Clinical Oncologists of Nigeria (ARCON) leadership who evaluated it for face validity, adequacy, and feasibility. After validity checks, ARCON leadership sent the questionnaire to 16 participants who gave their responses. The responses were analyzed. After this pilot study, the following edits were made to the questionnaire: five^5^ new items to measure satisfaction with delivering clinical oncologist (CO) care and a new item on the level of CO practice was added to the questionnaire. Additionally, items number 15 and 16 measuring clinical oncology workload were changed from hours to minutes. Only nine items measuring CO workload were adapted from global oncology workload survey (GOWS) after the pilot study. Other items on the GOWS were dropped as they were irrelevant to this study. Responses from the pilot were not included in the main study because of the edits.
